# A single-center study of prognostic impact and treatment implications of cribriform pattern in prostate cancer

**DOI:** 10.1007/s12672-026-05817-0

**Published:** 2026-08-24

**Authors:** A. Handke, M. Dellino, C. Orf, N. Wang, C. Darr, P. Bach, F. Roghmann, K. H. Tully

**Affiliations:** 1 Department of Urology and Neurourology, Marien Hospital Herne, Ruhr-University Bochum, Hölkeskampring 40, 44625 Herne, Germany; 2 Department of Urology, Brixen Public Hospital, Brixen, Italy; 3 Department of Urology, University Hospital Essen, Essen, Germany

**Keywords:** Cribriform prostate cancer, High-risk disease, Radical prostatectomy, Oncologic outcomes, Systemic therapy

## Abstract

**Introduction:**

Cribriform prostate cancer (cPCa) is increasingly recognized for its aggressive behavior and poor prognosis compared to conventional acinar adenocarcinoma. It is associated with adverse pathological features and excludes patients from active surveillance, highlighting the need for accurate identification. This study aimed to evaluate oncologic outcomes and characterize subsequent treatment patterns in a single-center cohort of patients with cribriform prostate cancer.

**Materials:**

We retrospectively analyzed 401 patients who underwent prostate biopsy and radical prostatectomy (RP) between 01/2020 and 12/2021 at a high-volume tertiary center. Of these, 112 patients had cPCa. Data on biopsy and prostatectomy specimens, post-treatment management, biochemical recurrence, and follow-up until August 2025 were collected. Cox regression was used to identify predictors of poor oncologic outcomes.

**Results:**

Of the 112 patients with cPCa, 66.1% had high-risk disease, and 30.4% had intermediate-risk disease. Biopsy detected 40 cases, with an additional 72 identified in the final post-RP specimen. At diagnosis, 6.3% had metastatic disease. At the time of surgery, 19.2% had lymph node metastases, and 16.4% had positive surgical margins. The median follow-up was 54 months. Univariable analysis showed that high Gleason score (≥ 8), lymph node positivity, and locally advanced stage were associated with decreased systemic therapy-free survival. In metastatic disease, no differences were found in therapy duration or time to castration-resistant prostate cancer.

**Conclusion:**

cPCa is an aggressive subtype with poor prognosis, characterized by high-risk features and early need for systemic therapy. These findings underscore the need for prospective studies exploring multimodal treatments for this high-risk population.

## Introduction

Prostate cancer (PCa) is among the most commonly diagnosed malignancies in men worldwide and a leading cause of cancer-related mortality. The majority of prostate cancers are acinar adenocarcinomas (PAA), characterized by small glandular formations. However, a subset exhibits a cribriform growth pattern (cPCa), which is increasingly recognized as a distinct histopathological entity with important clinical implications. cPCa-patterns have been acknowledged in current guidelines, underscoring their clinical significance, particularly in the context of treatment decisions, as their presence precludes active surveillance due to their association with aggressive disease behavior [1].

Our understanding of these less common histologic patterns in prostate cancer has significantly evolved in recent years. Compared with conventional acinar adenocarcinoma, cPCa exhibits distinct morphological and biological features that are associated with an unfavorable clinical course. Accurate recognition of these hallmarks is therefore paramount for clinical decision-making [2]. The presence of cribriform architecture has been linked to adverse pathological characteristics, including extraprostatic extension, seminal vesicle invasion, positive surgical margins, and lymph node metastases [3, 4]. Moreover, cPCa has been associated with an increased risk of disease progression, metastatic spread, and cancer-specific mortality [5–7].

However, despite its established prognostic relevance, the optimal clinical management of patients with cPCa remains incompletely defined. In particular, data regarding treatment strategies and long-term oncologic outcomes in contemporary clinical practice remain limited.

Given the distinct histopathological and clinical features of cPCa, accurate identification is crucial for guiding treatment decisions. This study aimed to evaluate oncologic outcomes, characterize treatment patterns, and assess the concordance between biopsy and radical prostatectomy regarding the detection of cribriform architecture in patients with cribriform prostate cancer treated at a high-volume tertiary referral center.

## Materials and methods

### Study design

We conducted a retrospective cohort study including patients diagnosed with cPCa at a single high-volume urological center between January 2020 and December 2021. Clinical data were collected and analyzed in accordance with institutional protocols. The study was approved by the institutional ethics committee (Ethikkommission Westfalen-Lippe; Approval number: 2025-163-f-S), and all procedures were carried out in accordance with the Declaration of Helsinki and relevant local regulations.

### Patient selection

Between January 2020 and December 2021, 401 consecutive patients underwent prostate biopsy followed by radical prostatectomy at our tertiary referral center. Among these, 112 patients were found to have histologically confirmed cPCa and were included in the present analysis. Patients were eligible if histopathological examination of the prostate biopsy and/or radical prostatectomy specimen demonstrated cribriform architecture. Patients with synchronous metastatic disease at diagnosis were included provided histological confirmation of cPCa was available. Patients with incomplete clinicopathological or follow-up data were excluded.

Histopathological findings from both biopsy and radical prostatectomy specimens were reviewed. The presence of cribriform architecture was recorded separately for biopsy and radical prostatectomy specimens to assess the detection rate of biopsy compared with final pathology. Whenever available, the proportion of the cribriform component was documented. Therapeutic data included all treatments administered after local therapy, time to biochemical recurrence, and follow-up through August 2025.

### Clinicopathological data collection

Baseline clinicopathological parameters were extracted from the institutional database. They included age at diagnosis, relevant comorbidities, preoperative PSA levels, clinical T-stage, biopsy Gleason score, and risk classification according to the EAU guidelines version applicable at the time of diagnosis. For patients undergoing RP, pathological findings were obtained from the final specimen, including tumor stage (pT), lymph node status (pN), surgical margin status, and Gleason score. Whenever available, the percentage of cribriform growth infiltration was assessed. Data regarding adjuvant, salvage, or later therapies (androgen deprivation therapy (ADT)), androgen receptor pathway inhibitors (ARPI), chemotherapy, or radiotherapy) were recorded.

### Outcome measures

The primary endpoint of this study was cancer-specific survival (CSS), defined as the interval between local treatment (radical prostatectomy or radiotherapy) and death from prostate cancer. Patients alive at last follow-up or who died of causes other than prostate cancer were censored. The secondary endpoints were systemic therapy-free survival (STFS), defined as the interval between local treatment and the initiation of any systemic therapy (ADT, ARPI, or chemotherapy) following biochemical recurrence or metachronous metastatic disease, and event-free survival (EFS) in the overall cohort, a composite endpoint comprising biochemical recurrence, a new diagnosis of metastatic disease, or death from any cause.

### Statistical analysis

Medians and interquartile ranges (IQRs) were generated for continuous variables, and frequencies and proportions for categorical variables. The chi-square test was used to compare categorical variables (e.g., the proportion of cPCa at diagnosis vs. at final pathology). Due to the limited sample size and number of events, separate univariable Cox proportional hazards regression models were used to identify predictors of event-free survival (EFS). Age and PSA at diagnosis, Gleason score (at the time of biopsy and at final histopathological review post radical prostatectomy), and pathological T-stage were examined as potential independent variables. Results were reported as hazard ratios (HR) with corresponding 95% confidence intervals (CI). Subsequently, Kaplan-Meier survival curves were fitted to visualize CSS, EFS, and STFS. For all time-dependent analyses, time zero was set at the date of diagnosis. Patients without systemic therapy or patients alive at the time of last follow-up were censored. Finally, unpaired t-tests were employed to examine differences in the mean duration of therapy in patients undergoing systemic therapy. All analyses were conducted using Stata (Version Stata/SE 15.1, Stata Corp LLC, TX, USA). A two-sided p-value < 0.05 was considered statistically significant.

## Results

### Patient baseline characteristics

A total of 112 patients with histologically confirmed cPCa were included in the analysis. At diagnosis, the median age was 66.5 years (IQR 60–71.5 years), and the median PSA was 10.9 ng/mL (IQR 6.2–17.9 ng/mL). The majority of patients presented with high-risk (*n* = 74, 66.1%) or intermediate-risk (*n* = 34, 30.4%) disease. At diagnosis, 7.1% (*n* = 8) of patients had synchronous metastatic disease. Further baseline characteristics are summarized in Table 1.

**Table 1:** Baseline characteristics of patients diagnosed with cribriforme prostate cancer (cPCa) (a) Patient and tumor-specific characteristics at the time of biopsy (*n* = 82) (b) Tumor-specific characteristics of patients with cPCa undergoing surgery (*n* = 104)

a) Patient and tumor-specific characteristics at the time of biopsy (*n* = 82)		
Median age, years (IQR*)		66.5 (60–71.5)
Median PSA^#^ at diagnosis, ng/ml (IQR)		10.9 (6.2–17.9)
Median prostate volume, cm^3^ (IQR)		36 (30–49)
Clinical T-stage (DRE^+^), n(%)	cT1c	46 (41.1)
	cT2a	19 (16.9)
	cT2b	15 (13.4)
	cT2c	13 (11.6)
	cT3	12 (10.7)
	Unknown	7 (6.3)
Gleason score at biopsy, n (%)	6	18 (16.1)
	7a	32 (28.6)
	7b	25 (22.3)
	8	18 (16.1)
	9	17 (15.2)
	10	2 (1.8)
D’Amico risk classification, n (%)	Low risk	4 (3.6)
	Intermediate risk	34 (30.4)
	High risk	74 (66.1)
Evidence of cPCa, n (%)	Yes	40 (35.7)
	No	72 (64.3)
Consecutive therapy, n (%)	Surgery	104 (92.9)
	Radiotherapy	0 (0.0)
	Systemic therapy (primary metastatic disease)	8 (7.1)
b) Tumor-specific characteristics of patients with cPCa undergoing surgery (*n* = 104)		
Gleason score at final pathology, n (%)	7a	48 (46.2)
	7b	34 (32.7)
	8	2 (1.9)
	9	19 (18.3)
	10	1 (0.9)
T-stage, n (%)	pT2c	42 (40.4)
	pT3a	37 (35.6)
	pT3b	23 (22.1)
	pT4	2 (1.9)
N-stage, n (%)	pN0	84 (80.8)
	pN1	18 (17.3)
	pN2	2 (1.9)
Resection status, n (%)	R0	87 (83.7)
	R+	17 (16.3)
Proportion of PDA on final histopathology, n(%)	≤ 5%	62 (59.6)
	6% − 49%	20 (19.2)
	≥ 50%	22 (21.2)

*IQR: Interquartile range; ^#^PSA: prostate specific antigen; ^+^DRE: digital-rectal examination

### Local treatment and pathologic findings

Among patients with non-metastatic disease (*n* = 104, 92.9%), all underwent radical prostatectomy. The majority of patients were diagnosed with Gleason 7a/7b (*n* = 83, 73.2%) or Gleason 9 (*n* = 19, 18.3%). 42 patients (40.2%) had localized disease (pT2c) on final histopathological evaluation. Extraprostatic extension (i.e., pT3a) and seminal vesicle invasion (i.e., pT3b) were observed in 35.6% (*n* = 37) and 22.1% (*n* = 23) of patients, respectively. Postoperative pathological analysis revealed lymph node-positive disease in 20 patients (19.2%) and positive surgical margins (PSM) in 17 patients (16.4%). In patients with pT2 and pT3a disease, only 7.1% (*n* = 3) and 8.1% (*n* = 3) were diagnosed with PSM, whereas in patients with pT3b disease, 43.5% (*n* = 10) of patients were found to have PSM after radical prostatectomy. At diagnosis, 40 patients (35.7%) had cPCa identified on biopsy, while an additional 72 patients (64.3%) were diagnosed on final pathology after radical prostatectomy.

### Oncological outcomes

**Fig. 1:**
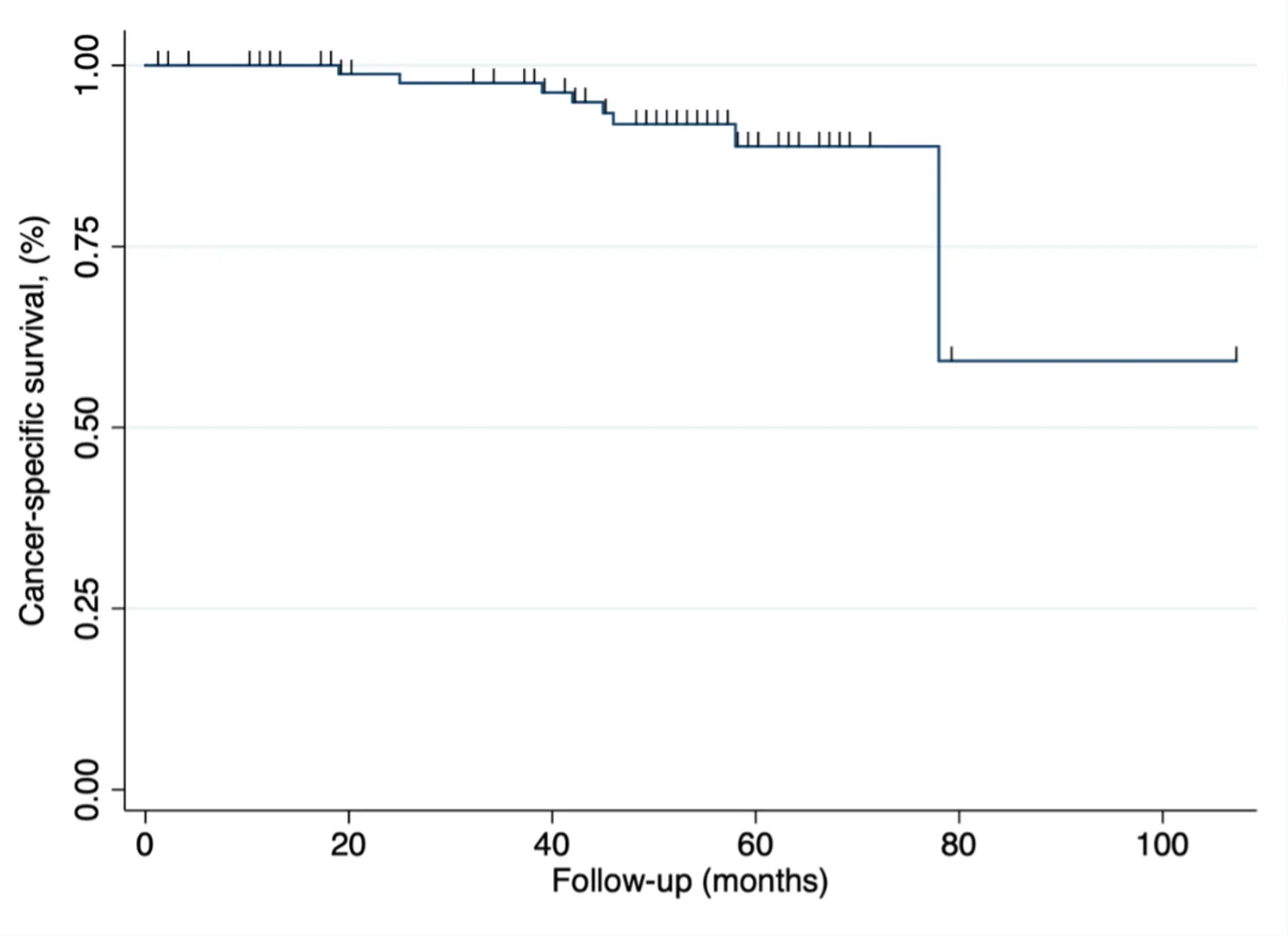
Cancer-specific survival in patients diagnosed with cribriform prostate cancer

**Fig. 2:**
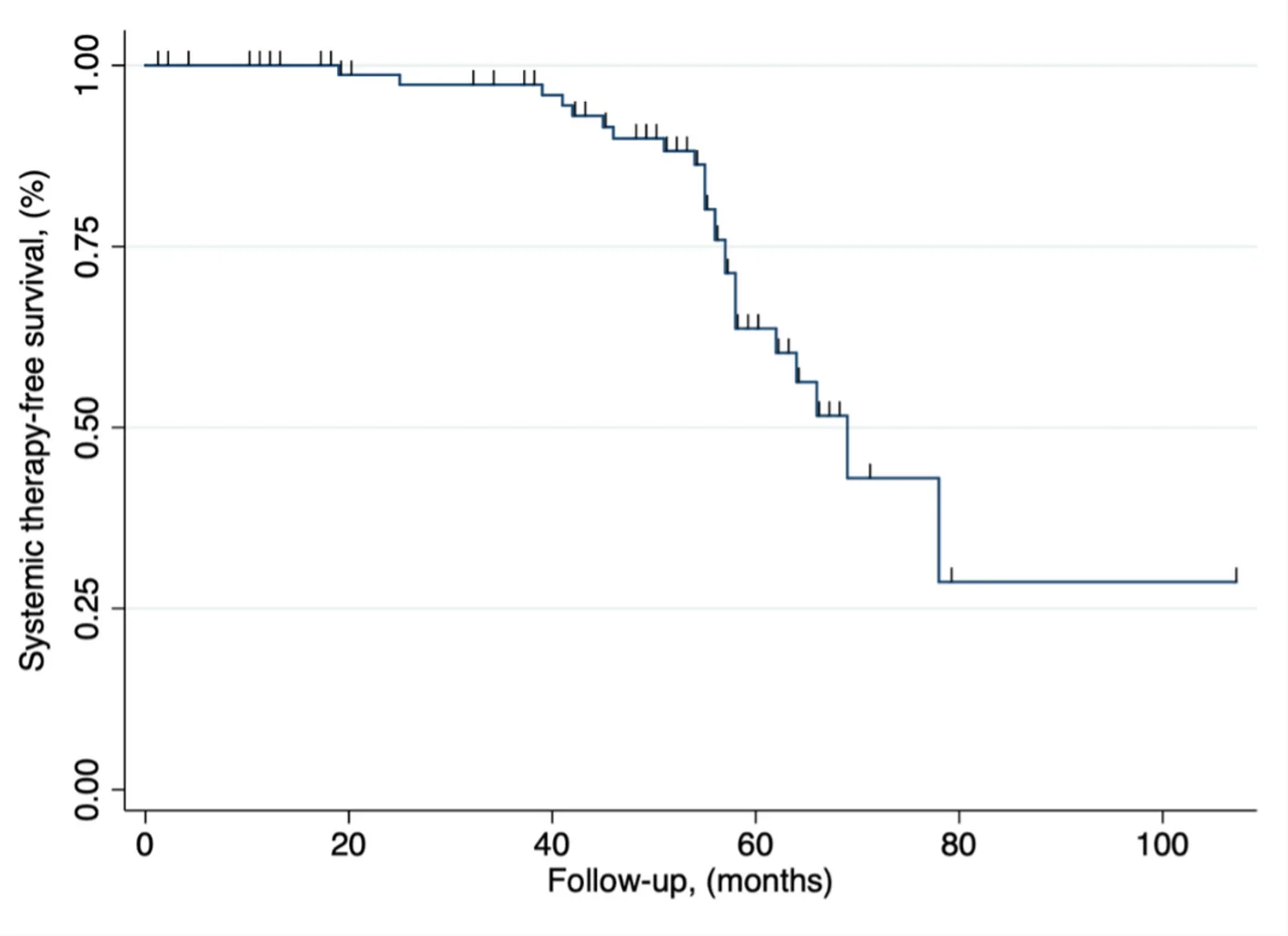
Time to systemic therapy for initially non-metastatic patients who had previously undergone radical prostatectomy

The median follow-up for the entire cohort was 54 months (IQR 41–58). Overall, 15 patients did not exceed the one-year follow-up landmark and were consequently considered as “lost to follow-up”. At the 5-year landmark, the median CSS had not been reached (Fig. 1). Eight patients (7,1%) died of prostate cancer during the observation period. During follow-up, 31 patients (27.7%) experienced an oncologic event, defined as cancer-specific death, development of metastatic disease, or biochemical recurrence. Among patients who initially underwent radical prostatectomy (*n* = 104), 24 patients (23.1%) experienced an event. The median time to systemic therapy for previously non-metastatic patients (*n* = 104, 92.9%) was 69 months (IQR 57 – not reached) (Fig. 2).

### Predictors of unfavorable outcomes

In the overall cohort, only a high Gleason Grade at the time of surgery (≥ 8) was significantly associated with an increased risk of cancer-specific death (HR 4.80, 95% CI 1.14–20.1, *p* = 0.032) (Table 2).

Among previously non-metastatic patients undergoing radical prostatectomy, univariable Cox regression analysis demonstrated that a high initial Gleason score (≥ 8) (HR 2.11, 95% CI 1.03–4.33, *p* = 0.041) and locally advanced disease stage (≥ pT3) (HR 3.36, 95% CI 1.36–8.27, *p* = 0.008) were associated with an increased risk of the composite endpoint of cancer-specific death, metastatic progression, or biochemical recurrence (Table 3a). No significant associations were observed for age, PSA at diagnosis, or surgical margin status.

In the same subgroup, locally advanced disease stage (≥ pT3) (HR 4.33, 95% CI 1.47–12.8, *p* = 0.008), lymph node-positive disease at radical prostatectomy (HR 2.23, 95% CI 1.11–4.50, *p* = 0.024), and a high Gleason score (≥ 8) at diagnosis (HR 3.21, 95% CI 1.45–7.12, *p* = 0.004) and at surgery (HR 2.78, 95% CI 1.26–6.14, *p* = 0.012) were associated with a shorter time to initiation of systemic therapy during follow-up (Table 3b).

**Table 2:** Cox proportional hazards regression analyses examining cancer-specific survival

		Hazard ratio	95% Confidence interval	*p*-value
T-stage	< pT3	REF	–	–
	≥pT3	2.44	0.48–12.4	0.284
N-stage	pN0	REF	–	–
	pN+	2.24	0.64–7.91	0.209
Gleason at biopsy	≤ 7b	REF	–	–
	≥ 8	3.50	0.84–14.6	0.087
Gleason score at final pathology	≤ 7b	REF	–	–
	≥ 8	4.80	1.14–20.1	0.032
PSA at diagnosis		0.98	0.92–1.05	0.547
Proportion of cPCa* on final histopathology	≤ 5%	REF	–	–
	6% − 49%	0.71	0.08–6.12	0.753
	≥ 50%	0.51	0.10–2.72	0.431
*cPCa: Cribriforme prostate cancer				

**Table 3:** Cox proportional hazards regression analyses examining event-free survival and systemic therapy-free survival in initially non-metastatic patients undergoing surgery (a) event-free survival (b) systemic therapy-free survival

		Hazard ratio	95% Confidence interval	*p*-value
a) Event-free survival				
T-stage	< pT3	REF	–	–
	≥pT3	3.36	1.36–8.27	0.008
N-stage	pN0	REF	–	–
	pN+	1.91	0.98–3.76	0.058
Gleason at biopsy	≤ 7b	REF	–	–
	≥ 8	2.11	1.03–4.33	0.041
Gleason score at final pathology	≤ 7b	REF	–	–
	≥ 8	1.97	0.95–4.11	0.069
PSA at diagnosis		1.01	0.99–1.03	0.254
Proportion of cPCa* on final histopathology	≤ 5%	REF	–	–
	6% − 49%	1.58	0.56–4.51	0.389
	≥ 50%	1.35	0.61–2.97	0.460
b) Systemic therapy-free survival				
T-stage	< pT3	REF	–	–
	≥pT3	4.33	1.47–12.8	0.008
N-stage	pN0	REF	–	–
	pN+	2.23	1.11–4.50	0.024
Gleason at biopsy	≤ 7b	REF	–	–
	≥ 8	3.21	1.45–7.12	0.004
Gleason score at final pathology	≤ 7b	REF	–	–
	≥ 8	2.78	1.26–6.14	0.012
PSA at diagnosis		1.01	0.99–1.04	0.362
Proportion of cPCa* on final histopathology	≤ 5%	REF	–	–
	6% − 49%	1.32	0.42–4.14	0.639
	≥ 50%	1.72	0.73–4.05	0.216
*cPCa: Cribriforme prostate cancer				

### Systemic therapy and treatment response

During follow-up, 24.1% (*n* = 27) of patients received systemic therapy. Among patients who developed metastatic disease, first-line systemic treatment consisted of ADT monotherapy (*n* = 19), androgen receptor pathway inhibitors (ARPI; *n* = 7), or docetaxel (*n* = 1). No significant differences were observed regarding treatment duration between first-line treatment modalities (*p* = 0.179).

The median time to castration-resistant prostate cancer (CRPC) among patients receiving systemic therapy for metastatic disease was 5.5 months (IQR 2–10), with no significant differences according to first-line treatment modality.

## Discussion

cPCa is a distinct, aggressive histologic subtype often associated with a poor prognosis. In this cohort, most patients presented with high-risk or advanced disease, with frequent lymph node metastases and PSM. High Gleason scores (≥ 8), advanced T-stage (≥ T3). Beyond confirming the aggressive biological behavior of cPCa, our study highlights the substantial discrepancy between biopsy and radical prostatectomy in the detection of cribriform architecture. While only 35.7% of cases were identified on biopsy, nearly two-thirds were detected only in the final radical prostatectomy specimen. Given the well-established prognostic significance of cribriform growth, this finding underscores the limitations of current preoperative diagnostics and their potential impact on risk stratification and treatment planning. Furthermore, high Gleason score (≥ 8), locally advanced disease, and lymph node positivity emerged as the strongest predictors of an earlier need for systemic therapy, suggesting that surgery alone may be insufficient for a subset of patients with cPCa. Overall, these findings are consistent with previous studies demonstrating that cribriform architecture is associated with biochemical recurrence, metastatic progression, and cancer-specific mortality, even after adjustment for Gleason Group and pathological stage [8–10].

cPCa is also consistently linked to adverse pathological features, including extraprostatic extension and seminal vesicle invasion, reinforcing its aggressive biology beyond conventional Gleason grading [4, 5]. Evidence regarding PSM is more heterogeneous, as some studies report higher PSM rates in cPCa, particularly in univariable analyses, likely reflecting its infiltrative growth pattern and association with locally advanced disease. In contrast, others do not find an independent association after adjusting for pathological stage and surgical factors [4, 5, 11, 12]. This heterogeneity is also reflected in our cohort, where PSM rates increased markedly with advancing pathological stage (7.1% in pT2, 8.1% in pT3a, and 43.5% in pT3b disease), suggesting that the association between cPCa and margin positivity is largely mediated by advanced local tumor extension rather than cribriform architecture per se. Accordingly, studies focusing on primary tumor characteristics tend to demonstrate associations between cPCa and global pathological or oncologic outcomes, whereas margin-focused analyses emphasize tumor grade and margin extent over architectural patterns [4, 5, 11, 12]. Overall, literature supports a strong link between cribriform growth, advanced pathological stage, and increased recurrence risk, although its independent prognostic impact at the surgical margin remains variable.

In our cohort, the prevalence of lymph-node positive disease was 7.1%. In contrast, the exact prevalence of synchronous metastatic disease among cribriform-positive prostate cancers is not well defined in the literature, as most studies do not report metastasis rates stratified by cribriform status across unselected cohorts. Instead, reported rates vary substantially depending on endpoint definition, cohort composition, and staging modality, complicating direct comparisons across studies. For example, in a multicenter cohort with favorable-risk Gleason Group (GG) 2 from radical prostatectomy, the absolute rate of synchronous nodal disease was low (7.4%). Yet, cribriform or intraductal carcinoma was present in all pN1 cases compared with only 51% of matched pN0 controls, underscoring the disproportionate association of cribriform architecture with early metastatic spread despite low baseline risk [13]. Conversely, a single-center study of high-grade biopsy cases using contemporary staging demonstrated markedly higher rates of de novo metastatic disease, with cribriform pattern and semiquantitative cribriform burden independently associated with metastatic presentation. However, discrimination remained moderate (AUC 0.70), reflecting intentional enrichment for aggressive disease rather than population-level prevalence [14].

In contrast, a post-RP cohort staged with Prostate-Specific Membrane Antigen Positron Emission Tomography/Computed Tomography (PSMA-PET/CT) showed that approximately 33% of included patients demonstrated lymphatic spread, highlighting that advanced imaging primarily refines metastatic detection rather than inflating population-level metastatic prevalence [15]. Taken together, these data indicate that cribriform carcinomas are consistently overrepresented among patients presenting with synchronous nodal or distant metastases. In contrast, the reported absolute rates of metastasis are highly dependent on cohort selection, endpoint definition, and staging approach, supporting the concept of early systemic disease biology in cPCa.

Across most contemporary series, prostate biopsy demonstrates limited sensitivity but high specificity for detecting cribriform carcinoma, with approximately half of cribriform patterns present in RP specimens missed at biopsy, while specificity typically exceeds 85–90% [16]. Consequently, cribriform architecture identified on biopsy is almost invariably confirmed at RP, whereas a negative biopsy finding does not reliably exclude its presence. This pattern is closely reflected in our cohort, in which only 35.7% of cribriform cases were detected on biopsy, while the majority (64.3%) were identified exclusively on final pathology. This discrepancy carries direct clinical implications: in current practice, treatment decisions, including the choice between radical prostatectomy and radiotherapy, are predominantly based on biopsy findings. Patients proceeding to radiotherapy without surgical specimen assessment may therefore harbour undetected cribriform architecture that remains unaccounted for in risk stratification and treatment planning. The considerable variability in reported detection rates across studies is attributable primarily to differences in biopsy quality, pathology review practices, and cohort composition [4, 16]. In particular, extremely low sensitivities described in some series can be explained by heterogeneous sampling strategies and non-centralized biopsy interpretation, especially when contrasted with expert central review of RP specimens. This combination amplifies both undersampling and interobserver variability [4]. In contrast, studies restricted to higher-risk or cribriform-enriched cohorts consistently report moderately higher sensitivities of approximately 40–56%, reflecting intentional preselection for aggressive disease rather than actual improvements in diagnostic performance [17].

The incremental value of imaging-guided targeting appears limited. Although multiparametric magnetic resonance imaging (mpMRI)-targeted biopsies modestly increase sensitivity in some cohorts, a substantial proportion of cribriform lesions remains undetected, likely due to the limited MRI conspicuity of many cribriform foci. Other contemporary series report no clear benefit of MRI-fusion techniques over systematic sampling [17–19]. This is in line with our findings, as in the present cohort, all patients received an mpMRI before which was interpreted by a dedicated uro-radiologist. Similarly, grade group-restricted analyses, including Gleason GG 2 focused cohorts, demonstrate sensitivity ranges comparable to unselected populations, indicating that biopsy underdiagnosis is not confined to low-grade disease, even though larger cribriform patterns are somewhat more likely to be sampled [16].

A further clinically relevant finding of the present analysis concerns the quantitative extent of cribriform growth. The proportional cribriform area at RP was not a significant predictor of adverse oncological outcomes in our cohort. This suggests that it may not be the quantity of cribriform architecture that determines prognosis, but rather its mere presence. If confirmed in larger prospective series, this would have meaningful clinical consequences as a patient with even a minor cribriform component could carry a clinically relevant risk that currently escapes reliable preoperative detection. In other words, we may already possess a prognostically important risk factor yet lack the diagnostic tools to capture it consistently before treatment decisions are made.

Overall, these data indicate that prostate biopsy systematically underestimates the true prevalence of cribriform architecture observed at RP, with differences across studies primarily driven by biopsy technique, imaging characteristics, pathology expertise, and cohort selection rather than actual biological variation. In this context, the high rate of postoperative upstaging observed in our cohort further supports the need for improved diagnostic strategies, including optimized biopsy approaches and standardized pathological reporting, to ensure timely identification of patients with high-risk disease. The combination of systematic biopsy underdetection and the prognostic relevance of even minimal cribriform involvement creates a diagnostic gap that current standard-of-care approaches including mpMRI-targeted biopsy do not reliably close. Addressing this gap remains one of the central unresolved challenges in the preoperative risk stratification of prostate cancer.

Our data further support the concept that cPCa should be managed as a high-risk prostate cancer subtype, regardless of the relative proportion of cribriform involvement. Several studies have demonstrated that even a minor cribriform component is associated with significantly worse outcomes compared to non-cribriform tumors [4, 5, 13]. In line with current guideline recommendations, patients with cPCa should be excluded from active surveillance protocols due to the substantial risk of early progression and metastasis [1]. Instead, definitive local therapy combined with risk-adapted treatment intensification should be considered, for example, with early adjuvant radiotherapy or systemic therapy, which may improve disease control in high-risk pathological subgroups, including those with cribriform architecture, although prospective data remain limited.

Several limitations of our study must be acknowledged. First, as a retrospective singlecenter analysis, treatment selection bias and heterogeneity in management approaches are unavoidable. Additionally, the number of patients receiving systemic therapy and the lack of data granularity in this setting may limit the informativeness of this analysis. Despite the high volume of our center, the overall cohort remains relatively small, further highlighting the rarity of this histologic subtype and limiting our analysis to univariable regression models. Further, detailed data on the percentage of cribriform growth infiltration at the time of biopsy were not available in the majority of cases, which limits biological correlative analysis. Furthermore, risk classification was based on the EAU guideline recommendations applicable during the study period (2020–2021). Since more recent guideline updates have modified the role of clinical staging, including DRE, some patients might be classified differently according to current recommendations. Additionally, this study did not include a direct internal comparison cohort of patients with conventional acinar adenocarcinoma, as the detailed manual data extraction required for such an analysis was beyond the scope of the present work. However, the independent adverse prognostic impact of cribriform histology compared with conventional adenocarcinoma has already been robustly established in multiple external cohorts, even after adjustment for Gleason Group and pathological stage [8–10]. Finally, follow-up remains relatively short for a disease with a potentially long natural history, and specific outcome measures (e.g., cancer-specific survival stratified by systemic therapy subtype) could not be robustly assessed.

## Conclusion

Cribriform prostate cancer represents a distinct and aggressive histologic subtype associated with advanced pathological features, including higher Gleason scores, extraprostatic extension, and lymph node metastases. In our cohort, high Gleason score, locally advanced stage, and nodal involvement were strong predictors of early systemic therapy, highlighting the limitations of local treatment alone. Biopsy underestimates the presence of cribriform architecture in the majority of cases, underscoring the importance of optimized sampling and standardized pathological reporting. These findings support managing cPCa as a high-risk entity, warranting exclusion from active surveillance and consideration of early treatment intensification. At the same time, prospective studies are needed to define optimal multimodal strategies for the future.

## Data Availability

The datasets generated and analyzed during the current study are not publicly available due to institutional data protection regulations and patient privacy restrictions but are available from the corresponding author on reasonable request and with permission of the institutional review board.
